# RNA m5C methylation modification: a potential therapeutic target for SARS-CoV-2-associated myocarditis

**DOI:** 10.3389/fimmu.2024.1380697

**Published:** 2024-04-23

**Authors:** Yan Xiong, Yanan Li, Weiwei Qian, Qing Zhang

**Affiliations:** ^1^ Department of Cardiology, West China Hospital, Sichuan University, Chengdu, Sichuan, China; ^2^ Department of Cardiology, Sichuan Academy of Medical Sciences & Sichuan Provincial People’s Hospital, Chengdu, Sichuan, China; ^3^ Emergency Department, Shangjinnanfu Hospital, West China Hospital, Sichuan University, Chengdu, Sichuan, China; ^4^ Department of Emergency Medicine, Laboratory of Emergency Medicine, West China Hospital, and Disaster Medical Center, Sichuan University, Chengdu, Sichuan, China

**Keywords:** COVID-19, RNA m5C methylation modification, myocarditis, NSun2, innate immunity

## Abstract

The Corona Virus Disease (COVID-19), caused by the Severe Acute Respiratory Syndrome Coronavirus-2 (SARS-CoV-2), has quickly spread worldwide and resulted in significant morbidity and mortality. Although most infections are mild, some patients can also develop severe and fatal myocarditis. In eukaryotic RNAs, 5-methylcytosine (m5C) is a common kind of post-transcriptional modification, which is involved in regulating various biological processes (such as RNA export, translation, and stability maintenance). With the rapid development of m5C modification detection technology, studies related to viral m5C modification are ever-increasing. These studies have revealed that m5C modification plays an important role in various stages of viral replication, including transcription and translation. According to recent studies, m5C methylation modification can regulate SARS-CoV-2 infection by modulating innate immune signaling pathways. However, the specific role of m5C modification in SARS-CoV-2-induced myocarditis remains unclear. Therefore, this review aims to provide insights into the molecular mechanisms of m5C methylation in SARS-CoV-2 infection. Moreover, the regulatory role of NSUN2 in viral infection and host innate immune response was also highlighted. This review may provide new directions for developing therapeutic strategies for SARS-CoV-2-associated myocarditis.

## 1 Introduction

Viral myocarditis is a serious heart disease caused by viral infection, which poses a major threat to human health, especially to children and young men. Previous research has reported up to 20 viruses linked to viral myocarditis, and the most prevalent are influenza virus, enterovirus, adenovirus, human herpesvirus, cytomegalovirus, Epstein-Barr virus (EBV), hepatitis C virus, and parvovirus B19 (1, 2). The emergence of a novel coronavirus in 2019, the Severe Acute Respiratory Syndrome Coronavirus-2 (SARS-CoV-2), has resulted in a global pandemic of the Corona Virus Disease (COVID-19). There is a growing interest in SARS-CoV-2-associated myocarditis due to the increase in respiratory and cardiovascular complications-associated deaths (3). SARS-CoV-2-induced myocarditis often occurs in hospitalized patients who are seriously or critically ill. But if it attacks the heart, patients may experience severe myocardial damage, shock, and even death (3, 4).

RNA methylation, a widespread post-translational modification of RNA, can regulate a wide range of biological processes (5). Among the more than 150 types of RNA methylation, 5-methylcytosine (m5C) is one of the important methylation modification types on eukaryotic messenger RNAs (mRNAs) (i.e., the fifth carbon atom of cytosine is methylated) (6). The development of next-generation sequencing approaches [such as RNA bisulfite sequencing, m5C RNA immunoprecipitation sequencing (m5C-RIP-seq), 5-azacytidine-mediated RNA immunoprecipitation sequencing (Aza-IP-seq), and methylation individual-nucleotide-resolution crosslinking and immunoprecipitation sequencing (miCLIP-seq)] has enabled transcriptome-wide mapping of m5C methylation (7–9). Moreover, it has been demonstrated that m5C methylation affects several cellular processes, including nuclear RNA export, mRNA translation, cell cycle control, cell differentiation, proliferation, and development (10). In recent years, the successful mapping of m5C modifications of RNA in selected retroviruses, DNA viruses, flaviviruses, and coronaviruses has laid the foundation for exploring the role of m5C methylation in viruses (11–17).

The myocardial injuries caused by SARS-CoV-2 and its multiple variants are gradually gaining attention as they have normalized (18). Meanwhile, m5C methylation has attracted much attention to its role in regulating many cellular processes. Therefore, this review aims to delve into the pathogenesis of SARS-CoV-2-induced myocarditis and the underlying mechanisms of RNA m5C methylation, providing clues for the potential association between SARS-CoV-2-induced myocarditis and m5C methylation. In addition, how m5C affected the viral life cycle and the innate antiviral immune response were also explored. Shortly, this review revealed the possible role of m5C methylation in SARS-CoV-2-associated myocarditis as well as provided the basis and insights for exploring new therapeutic avenues.

## 2 Biological functions of m5C methylation

**Figure 1:**
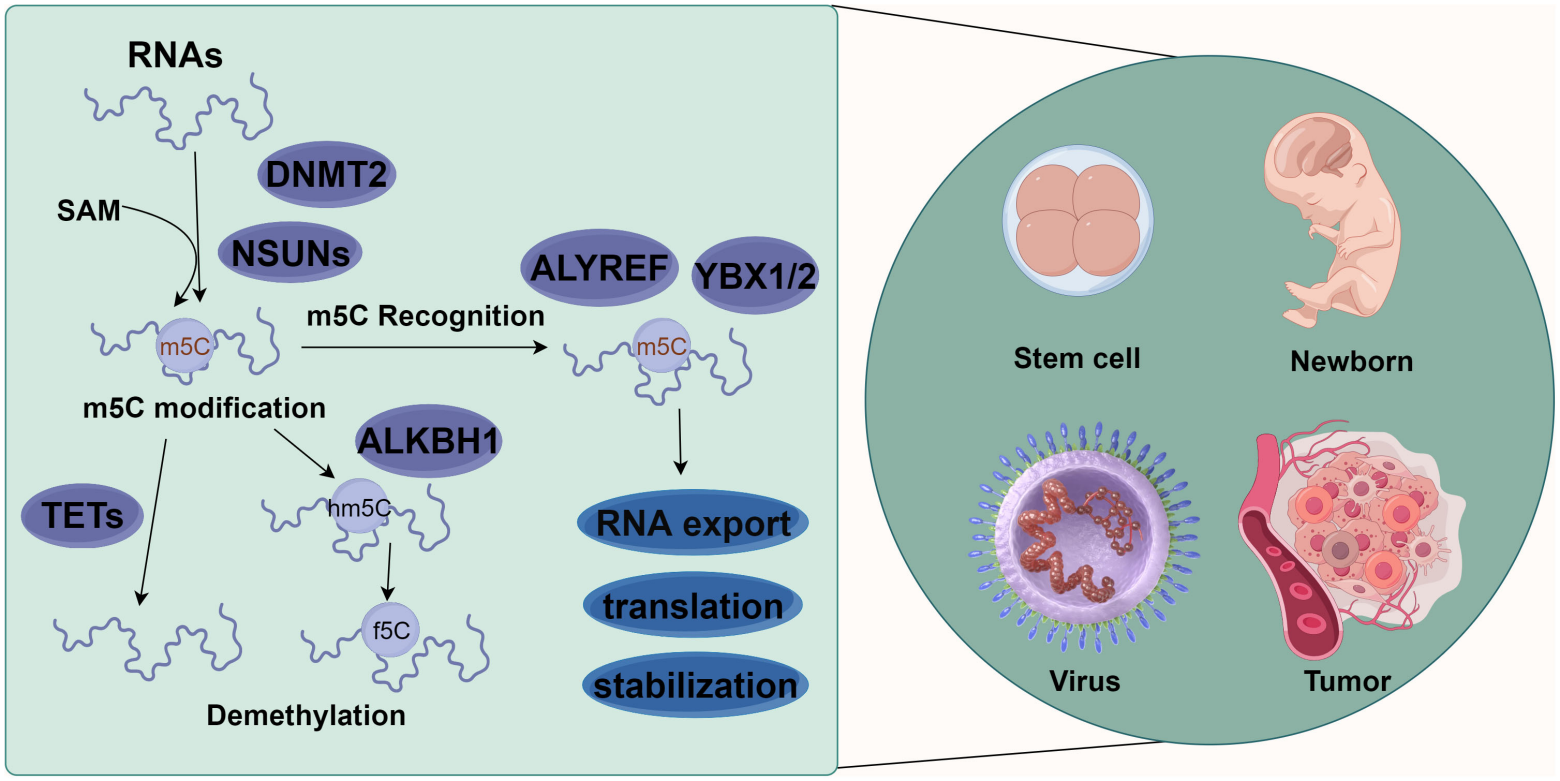
Molecular mechanism of m5C methylation modification. m5C methylation modification is involved in various biological functions, such as stem cell differentiation, newborn growth, virus replication, and tumor occurrence and development. Different types of RNAs (such as tRNA, rRNA, mRNA, vtRNA, snRNA, snoRNA, and miRNA) methylate the 5th carbon atom of cytosine (C) under the action of methylases NSUN1-7 and DNMT2. Meanwhile, demethylases TET1-3 and ALKBH1 could reverse this methylation. These methylated RNAs can affect the export, transport, translation, and stabilization of RNAs via the action of recognition proteins such as ALYREF, YBX1, and YBX2 (By figdraw.).

As an important form of post-transcriptional modification of RNA, m5C is ubiquitous in eukaryotic mRNA (19), transfer RNA (tRNA) (20), and ribosomal RNA (rRNA) (21), accounting for 0.05% of all the C in eukaryotic mRNA. It is involved in the regulation of metabolic processes such as RNA export, translation, and stability maintenance (10, 22). This dynamically reversible modification is catalytically generated by methyltransferases, removed by demethylases, and recognized by specific readers. On the other hand, m5C is also involved in regulating many physiological and pathological processes such as embryonic development (19, 23, 24), tumor development (25, 26), viral replication (22, 27), and stem cell differentiation (28–31) (**Figure 1**).

### 2.1 m5C methyltransferases

RNA m5C modifications are catalyzed by RNA m5C methyltransferases, with S-adenosyl-l-methionine (SAM) as a methyl donor for methylation modification at the 5th carbon atom of cytosine (32). Various m5C-specific methyltransferases have been identified and characterized, including members of the NOL1/NOP2/SUN domain (NSUN) family of proteins (consisting of seven members in humans, NSUN1-7) and the DNA methyltransferase (DNMT) homologue DNMT2 (33). Within the NSUN family, NSUN2 is one of the most characterized members and a major m5C “writer” (34). m5C methylation has been found in diverse RNA species, including tRNAs (6), rRNAs (35), mRNAs (25), vault RNAs (vtRNAs) (36), small nuclear RNAs (snRNAs) (22), small nucleolar RNAs (snoRNAs) (37), microRNAs (miRNAs) (38), and viral RNAs (27). m5C modification, a common methylation modification on eukaryotic mRNAs, is more likely to occur in GC dibase-enriched regions, such as near the translation initiation site/start codon of the coding regions (CDS) (19).

### 2.2 m5C demethylases

The identified m5C-modifying demethylases include the Ten-eleven translocation (TET) protein family (TET1-3) and ALKBH1 (AlkB Homolog 1, Histone H2A Dioxygenase). TETs are α-ketoglutarate- and Fe^2+^-dependent dioxygenases responsible for catalyzing m5C demethylation and generating 5-hydroxymethylcytosine (hm5C) (39). In contrast to TETs, ALKBH1 catalyzes the 1^st^ step of m5C demethylation by converting m5C to 5-hydroxymethylcytosine (hm5C) and further oxidizing 5hmC to 5-formylcytosine (f5C). ALKBH1 has also been reported to be mainly involved in regulating the demethylation of tRNAs, including mt-tRNA Leu and mt-tRNA Met (9, 40).

### 2.3 m5C methylation recognition proteins

The identified m5C methylation recognition proteins of eukaryotic mRNAs include Aly/REF export factor (ALYREF) (41), Fragile X mental retardation protein (FMRP) (42), Y-box binding protein (YBX)1, and YBX2 (43). ALYREF and FMRP are predominantly present in the nucleus, whereas YBX1 and YBX2 are in the cytoplasm (34). Accumulating studies have provided evidence for the tight association between these recognition proteins and m5C modifications. For example, ALYREF specifically recognizes m5C modifications on mRNAs to facilitate their export from the nucleus through lysine K171 (44). YBX1 recognizes m5C modifications on mRNAs through an indole ring of W65 within its cold-shock domain to maintain its stability (26). YBX2 binds with m5C-modified mRNAs through the W100 of the cold-shock domain to facilitate liquid-liquid phase separation (43). FMRP participates in the TET1-mediated R-loop demethylation via the recruitment of DNMT2, ensuring the effective repair of DNA damage (42).

## 3 Molecular mechanism of SARS-CoV-2-associated myocarditis

### 3.1 Manner of SARS-CoV-2 infecting cardiomyocytes

**Figure 2:**
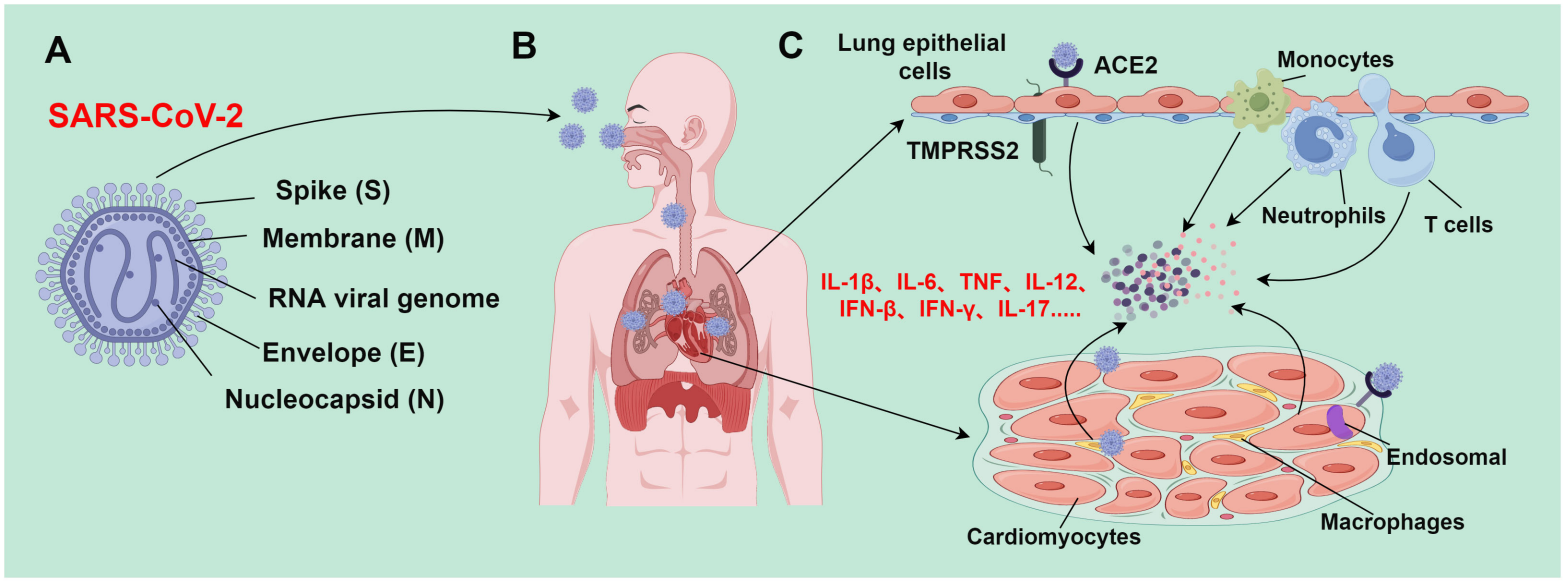
Mechanism of SARS-CoV-2-induced tissue damage (By figdraw.). **(A)** SARS-CoV-2 is an enveloped, positive sense, and single-stranded RNA virus with a genome of about 30,000 nucleotides, which encodes major structural proteins such as spike protein (S), nucleocapsid protein (N), membrane protein (M), and envelope protein (E); **(B)** SARS-CoV-2 is transmitted to various body organs (such as the heart) mainly through the respiratory tract; **(C)** during epithelial infection, SARS-CoV-2 binds to ACE2 receptors; TMPRSS2 facilitates the virus to fuse with airway epithelial cells, leading to the release of viral genomic RNA into the cytoplasm. However, SARS-CoV-2 enters cardiomyocytes through endosomes-dependent mechanisms without requiring TMPRSS2. The SARS-CoV-2 infection process activates the immune system and triggers the release of a large number of inflammatory cytokines; these inflammatory cytokines subsequently recruit and activate more immune cells (such as monocytes, macrophages, neutrophils, and Th1 cells) to form a positive feedback loop. When the inflammation level reaches a certain threshold, inflammatory responses would be out of control, resulting in an inflammatory cascade. This inflammatory cascade can lead to inflammatory cell death (PANoptosis), and eventually trigger severe pathological changes in lung and heart tissues.

Over the past two decades, there has emerged 3 zoonotic coronaviruses: SARS-CoV, middle east respiratory syndrome coronavirus (MERS-CoV), and SARS-CoV-2. These coronaviruses spilled over from animal repositories into humans. Similar to other coronaviruses, SARS-CoV-2 is an enveloped, positive sense, and single-stranded RNA virus with a genome of about 30,000 nucleotides (45). Four major structural proteins [spike (S), nucleocapsid (N), membrane (M), and envelope (E) proteins] are encoded by the genome. Noticeably, a helical ribonucleoprotein complex is formed when the genomic RNA binds with the N protein (46) (**Figure 2A**). Accumulating studies have demonstrated the presence of SARS-CoV-2 viral protein and nucleic acid substances in myocardial cells of COVID-19 patients, even in patients without clinical symptoms of cardiac involvement (47–52). This indicates that SARS-CoV-2 infection can occur in myocardial cells. SARS-CoV-2 can infect myocardial cells through direct and indirect pathways.

#### 3.1.1 Direct infection

Angiotensin-converting enzyme 2 (ACE2) receptors are expressed in cardiomyocytes of normal hearts, and their expressions are higher in the heart than those in the lung (53). Additionally, the abnormal increase of ACE2 expression is tightly implicated in cardiomyocytes of patients with heart disease (54, 55). SARS-CoV-2 recognizes and binds to the ACE2 receptor into the cell through the S protein. ACE2 receptor is a membrane protein that can cleave angiotensin II (Ang II) into angiotensin 1-7 (Ang 1-7) to balance the adverse effects of the renin-angiotensin-aldosterone system (RAAS). In lung cells, type II transmembrane protease serine-2 (TMPRSS2) is involved in S protein cleavage, exposing the receptor-binding domain (RBD) of the S1 subunit and promoting its binding to ACE2 receptors (56, 57). Different from its infection mode in lung cell lines, SARS-CoV-2 enters cardiomyocytes through endosomes-dependent mechanisms, and TMPRSS2 is not required in this process (48, 49). The endosomes-dependent mechanism refers to the process of the virus releasing nucleic acid substances (such as genomic RNA) into the cytoplasm through intracellular endosomes after the virus enters the cell. The endosome-dependent mechanism usually occurs within specific cell types. In these types of cells, the virus does not directly fuse with the cell membrane but forms intercellular endosomes after entering the cell. This process is usually affected by an acidic environment. The acidic environment of the endosomes facilitates the virus to fuse with the endosomal membrane, and then release its genetic substances into the cytoplasm (58). Endosomes-dependent proteases (such as cathepsin and calpain) are highly expressed in cardiomyocytes at similar levels to those in the lung cells, which can compensate for S protein activation and mediate SARS-CoV-2 infection (49).

#### 3.1.2 Indirect infection

When the SARS-CoV-2 gene is overexpressed in lung epithelial cells, lung epithelial cells release extracellular vesicles that carry viral RNA. These extracellular vesicles can reach cardiomyocytes, and viral genes carried by these vesicles can cause inflammatory activation, thus indirectly infecting cardiomyocytes (59).

### 3.2 Mechanisms of cardiomyocyte damage caused by SARS-CoV-2

SARS-CoV-2 typically induces upper and lower respiratory tract infections, often accompanied by fever, cough, and loss of smell and taste. Most infections are mild, and up to 20-40% of patients are asymptomatic. However, severe and fatal conditions may also occur in some patients, such as systemic inflammation, tissue damage, acute respiratory distress syndrome, thromboembolic complications, cardiac injury, and/or cytokine storms (60) (**Figure 2B**). Based on current research advances, the mechanisms of myocardial injury in patients with SARS-CoV-2 can be summarized into the following 3 main points:

#### 3.2.1 SARS-CoV-2 infection can induce respiratory dysfunction and hypoxemia

SARS-CoV-2 mainly attacks airway epithelial cells, thereby invading host cells through interactions with ACE2 and TMPRSS2. This impairs the morphology and function of airway epithelial cells, including the formation of syncytial cells, disruption of cell junctions, and inordination of cilia array. These pathological changes directly weaken airway defenses, making the airways more susceptible to infection. Additionally, large amounts of mucus may be produced, which can block bronchi and small airways and interfere with normal respiratory gas exchange (61). During SARS-CoV-2 infection, the pulmonary vasculature will experience an inflammatory response that damages and malfunctions the endothelial cells therein. This may increase the risk of thrombosis and pulmonary embolism. Pathologic alterations (such as hemorrhagic spots, petechiae, and microthrombi) have been demonstrated in the lung tissues of SARS-CoV-2 patients. These alterations exacerbate air-blood barrier disruption and impair gas exchange (62). Long-term SARS-CoV-2 infection may induce pulmonary fibrosis, as manifested by interstitial fibrosis and thickening of the interlobular septa. This may be attributed to the extensive destruction of alveolar epithelial cells and pulmonary vascular endothelial cells caused by the virus (63). Moving forward, pulmonary fibrosis will result in long-term disruption of respiratory structure and function, ultimately leading to restrictive pulmonary deterioration and respiratory failure. Severe SARS-CoV-2 patients with lung damage exhibit high levels of cytokines [such as interleukin (IL)-6 and tumor necrosis factor (TNF)-α] (**Figure 2C**). Excessive release of these cytokines may affect myocardial contractile function through various signaling pathways, cause electrolyte disorders in the heart, induce myocardial cell apoptosis, and cause peripheral vasodilation, eventually leading to a sharp deterioration of heart function in patients (64).

#### 3.2.2 Cytokine storms triggered by an imbalance of helper T-cell (TH1) response will cause myocardial injury

SARS-CoV-2 infection induces the release of a large number of inflammatory cytokines, which recruit and activate more immune cells, finally establishing a positive feedback loop. When the inflammation level reaches a certain threshold, it triggers an out-of-control inflammatory response and sets off an inflammatory cascade. Enhanced TH1 responses may facilitate the release of large amounts of pro-inflammatory cytokines [such as IL-1β, IL-6, TNF, IL-12, interferon (IFN)-β, IFN-γ, and IL-17] from the over-activated immune system (65) (**Figure 2C**). The activated TH1 cells may contribute to cytokine storms by synergizing signaling and inducing inflammatory cell death (PANoptosis) (66–68). PANoptosis is a cell death pathway in which the overproduction of cytokines is mediated by inflammatory cell death. TNF and IFN-γ synergistically induce PANoptosis, which in turn triggers the production of more cytokines, thereby establishing a positive feedback loop. Cytokine storms may be directly related to PANoptosis. The synergistic action of TNF and IFN-γ signaling can lead to immune cell death via inducing PANoptosis. This may especially trigger injury in vital tissues such as the myocardium (69). Nevertheless, the mechanisms of cytokine storms are still controversial and uncertain, as various studies reach different conclusions. Therefore, more studies and evidence are needed to further clarify the detailed molecular mechanisms of SARS-CoV-2-triggered cytokine storms.

#### 3.2.3 Myocardial injury may be attributed to the reduced activity of the ACE2/angiotensin (1-7) axis that has a cardioprotective role in angiotensin II signaling

A previous study has shown that ACE2 and angiotensin (1-7) levels are reduced in autopsy heart samples from patients who tested positive (70). Despite the reduced ACE2 levels, the SARS-CoV-2 virus has a 10- to 20-fold higher affinity for binding to ACE2 receptors than SARS-CoV due to an amino acid difference in the S protein RBD. Overall, this may be one of the main reasons why SARS-CoV-2 is more infectious and pathogenic than SARS-CoV in the 2003 epidemic and is more likely to inflict myocardial injury (71–73).

## 4 SARS-CoV-2 interaction with innate immunity

### 4.1 Introduction to innate immunity

The innate immune response is the first barrier of the organism against invasion of exogenous microorganisms in the course of long-term evolution. Pattern recognition receptors (PRRs), an integral part of the host’s innate immune system, are essential for body resistance to pathogenic microbial infections. During viral entry and replication, the innate immune system can sense viral components. For example, once entering the cytoplasm, SARS-CoV-2 is thought to follow the same pathway as the other CoVs; the coronavirus genomic RNA is translated into two large polyproteins (pp1a and pp1ab), encoding 16 non-structural proteins (NSPs). These proteins promote the formation of viral replication-transcription complexes that can produce antisense negative strand templates from viral RNA (74). After that, NSP3 and NSP4 reorganize membranes originating from the endoplasmic reticulum (ER) and Golgi apparatus to generate double-membrane vesicles (DMV) that separate viral replication and transcription from host sensor detection (74–76). The S, E, and M proteins of the SARS-CoV-2 virus are exposed to host cell surface sensors during their binding, and host cytoplasmic sensors can detect viral proteins and nucleic acids before they are segregated by the NSP (76).

Innate immune cells [including macrophages, monocytes, dendritic cells (DCs), neutrophils, and innate lymphocytes (ILCs) such as natural killer (NK) cells] are equipped with a set of PRRs that can recognize pathogen-associated molecular patterns (PAMPs) or damage-associated molecular patterns (DAMPs) to induce inflammatory signaling pathways and immune responses (77, 78). Toll-like receptors (TLRs) (79), retinoic acid-inducible gene I (RIG-I)-like receptors (RLRs) (80), nucleotide-binding oligomerization domain (NOD)-like receptors (NLRs) (81), C-type lectin receptors (82) and Absent in melanoma 2 (AIM2)-like receptors are five main PRR families. Signaling through these receptors in innate immune cells in response to pathogens, PAMP or DAMP, triggers the production of inflammatory cytokines and chemokines while inducing cell death to clear the infected cells (77, 78). To date, several PRRs, especially TLRs, have been shown to activate their signaling pathways in response to SARS-CoV-2-induced myocarditis.

### 4.2 Dual role of innate immunity in SARS-CoV-2-associated myocarditis

**Figure 3:**
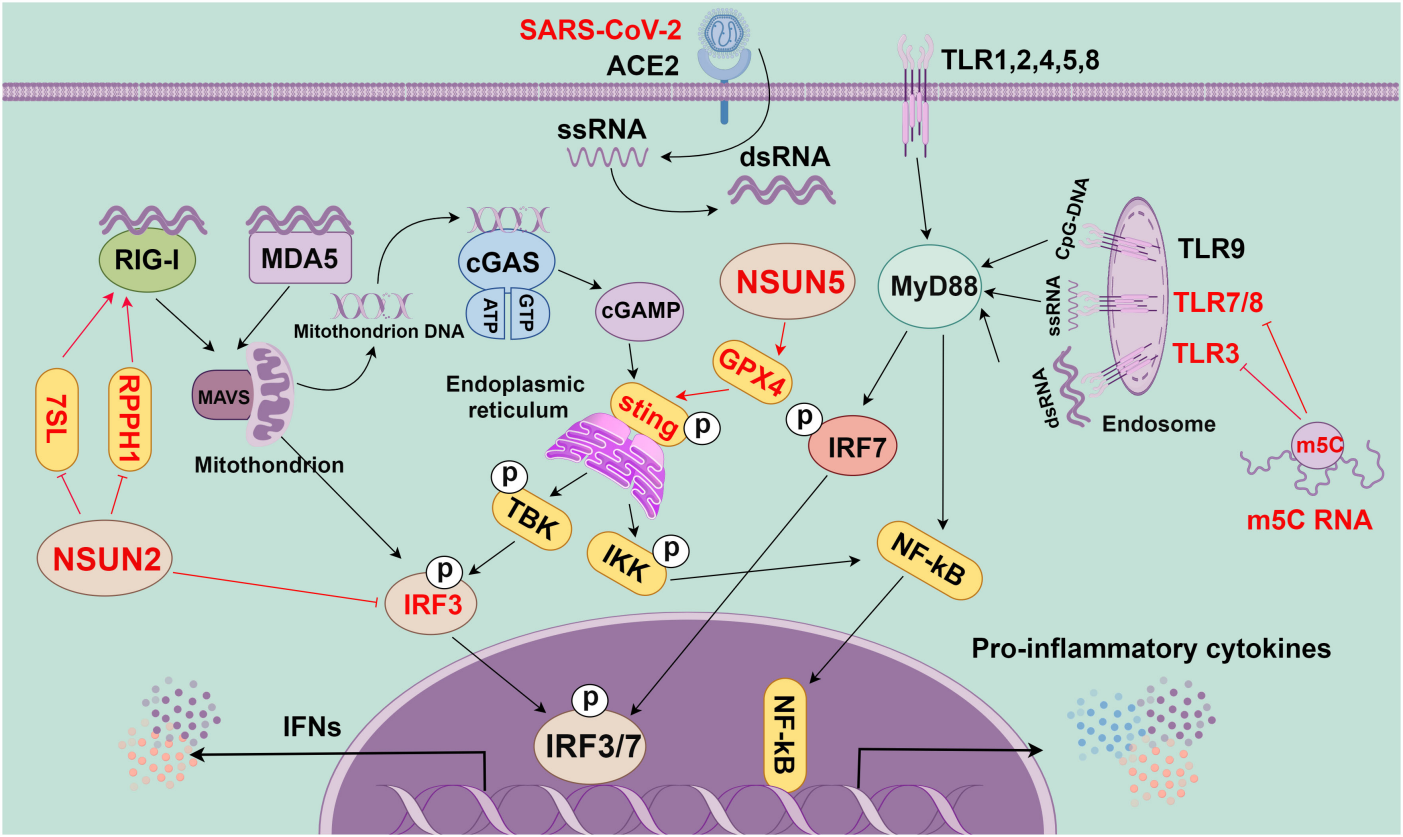
The role of m5C methylation in innate immunity (By figdraw.). First, the RNA of SARS-CoV-2 is reverse-transcribed into complementary RNA strands within the cell through the action of viral protein replicase and transcriptase to form a double-stranded RNA structure. This double-stranded RNA is crucial in viral replication and transcription, as it can activate the RNA sensing mechanism within the host cell to trigger the immune response, as manifested by the production of INFs and other inflammatory factors. In the innate immune system, toll-like receptor (TLR), cGAS, and RIG-I-like receptors (RLRs) are key pattern recognition receptors (PRRs). They are responsible for recognizing various invading pathogens, RNA, and DNA, as well as triggering immune responses. TLR is a crucial PRR in the innate immune system, and different TLRs can recognize different invading pathogens, dsRNA, ssRNA, and CpG-DNA. They activate the intracellular pathways and transcription factors (such as IRF7 and NF-kB) through adaptor proteins (such as MyD88) to trigger the immune response. cGAS, a cytoplasmic PRR, and cytoplasmic DNA sensor can recognize mitochondrial DNA in the cytoplasm to activate STING, which can recruit TBK1 and IKK and then activate IRF3 and NF-kB, eventually causing the release of inflammatory cytokines and IFNs. RLRs, a class of intracellular PRRs, mainly recognize viral RNA through RIG-I or MDA5 and transduce signals through MAVS to activate IRF3, thereby eventually leading to the release of IFNs. m5C modification plays a crucial role in these signaling pathways. Specifically, m5C-modified RNA can inhibit the activity of signaling activated by TLR3, TLR7, and TLR8. Additionally, NSUN5 promotes the activation of the cGAS-STING signaling pathway through methylating GPX4 mRNA. Meanwhile, NSUN2 can inhibit the host RNA polymerase III (Pol III)-transcribed non-coding RNA (ncRNA), especially RPPH1 and 7SL RNA, thereby inhibiting the RIG-mediated IFN response. All in all, m5C methylation modification plays a regulatory role in the innate immune signaling pathway and is essential for regulating the immune response.

SARS-CoV-2 infection triggers the complex and sophisticated response of the host’s innate immune system, in which multiple signaling pathways and molecules are involved in a synergistic manner (**Figure 3**). In the human respiratory tract, TLRs are key receptors in the innate immune system that are extensively distributed throughout the respiratory tract. Different TLRs show heterogeneous expressions in innate immune cells. For example, TLR3 is abundant in NK cells, whereas TLR4 is common in macrophages (83). TLRs trigger inflammatory cytokine production by transducing signals mainly through two key adaptor molecules: myeloid differentiation primary response gene 88 (MyD88) and TIR domain-containing adaptor-inducing interferon-β (TRIF) (84, 85). TLR4 can specifically bind with MyD88 or TRIF and signaling to activate molecules [such as nuclear factor kappa B (NF-κB), mitogen-activated protein kinase (MAPK), and interferon regulatory factor (IRF)], thereby transcriptionally activating various pro-inflammatory cytokines (including TNF, IL-6, and IL-1) (86).

TLR2 has been extensively documented to play roles in SARS-CoV-2 infection. For example, in macrophages of TLR2-deficient mice and TLR2 inhibitors-treated humans, SARS-CoV-2-mediated proinflammatory signaling pathways and cytokine production were attenuated after stimulation with SARS-CoV-2 E protein (87). Moreover, there is experimental evidence for the *in vivo* induction of TLR2-dependent inflammation by the SARS-CoV-2 E protein. However, further studies are still needed to determine whether TLR2 binds directly to E proteins or other SARS-CoV-2 ligands. Regarding TLR3, it has been shown that the TLR3 signaling pathway plays a protective role against SARS-CoV-2 in the organism (79, 88). Nevertheless, little is known about the precise role of TLR3 in SARS-CoV-2 infection. Although several genetic analyses have pointed out an association between congenital TLR3 defect and disease severity, further validation is needed. In a clinical study of myocarditis, TLR1-TLR10 mRNA can be detected in normal peripheral blood T cells, but only TLR2-TLR5 and TLR9 expression could be detected at the protein level. However, in patients with myocarditis, TLR1, TLR2, and TLR7 are overexpressed at the mRNA level (89). These findings imply that TLRs might be implicated in the pathogenesis of myocarditis. Additionally, the activation of TLRs can induce the production of inflammatory factors [including IFN-γ, IL-6, and tumor growth factor (TGF)-β], stimulate the differentiation of natural cells into Th1 cells, and then aggravate the immune response as well as the pathophysiological process of myocarditis. TLR overactivation, T-cell-mediated myocardial cytotoxicity, and cytokine overproduction may be important factors in SARS-CoV-2-induced myocarditis (90).

Recently, multiple episodes of myocarditis have been observed following vaccination with the mRNA SARS-CoV-2, especially in young men. The proposed explanations for this phenomenon involve mechanisms such as molecular mimicry, overreaction of the immune system to the mRNA, and dysregulation of the immune system (91, 92). Altogether, upon viral entry into the myocardium, the recognition of TLRs stimulates the activation of NF-kB and the release of pro-inflammatory cytokines (such as IL-1, IL-6, IL-12, TNF-α, and IFN-γ). The release of these cytokines further attracts immune cells to eradicate viral pathogens. However, sustained release of pro-inflammatory cytokines and immune cells may also cause myocardial cell damage and subsequent myocardial dysfunction (4). This cascade of pathophysiologic processes may work together in SARS-CoV-2-induced myocarditis. However, in-depth studies are still needed to explore the exact mechanisms.

Additionally, the IFN pathway is triggered once single-stranded RNA produced by viral infection is sensed by intracellular RLRs [such as RIG-I and melanoma differentiation-associated protein 5 (MDA5)]. IFN production and release stimulate the downstream signaling through the IFN receptor, inducing the production of various interferon-stimulated genes (ISGs) with antiviral functions. These ISGs [including lymphocyte antigen 6 family member E (Ly6E), members of the IFIT family, and bone marrow stromal cell antigen 2 (BST2)] can limit viral replication (93). However, the fine-tuning of a type I interferon (IFN-I) is critical as both over-activation and under-activation of IFN signaling can be deleterious to the host. Additionally, the cyclic GMP–AMP synthase (cGAS)–stimulator of interferon genes (STING) signaling pathway is activated when cytoplasmic DNA is detected, which is critical for limiting the replication of DNA and RNA viruses after infection. However, the SARS-CoV-2 accessory proteins (ORF3a and 3CL) can antagonize the cGAS-STING signaling and inhibit antiviral immune responses (94, 95). Overall, SARS-CoV-2 infection triggers the activation of several innate immune signaling pathways, including TLR, RLR, NLR, and cGAS-STING; these pathways exert synergistic effects, which is critical to the host resistance to viral infection (**Figure 3**). However, more comprehensive studies are still needed to investigate the detailed mechanisms and inter-regulation of each signaling pathway in SARS-CoV-2-induced myocarditis.

## 5 Potential role of m5C methylation in regulating SARS-CoV-2 infection

### 5.1 m5C methylation and SARS-CoV-2 infection

RNA m5C methylation is an important form of RNA modification that has garnered significant interest since its first discovery in 1975 in the intracellular 26S RNA of Sindbis virus (96). It has been shown that m5C methylation is widely present in multiple viruses, such as murine leukemia virus (MLV), human immunodeficiency virus type 1 (HIV-1), and SARS-CoV. In MLV infections, NSUN2 can modulate m5C modification, which in turn affects gene expression and viral replication (97, 98). Similarly, in HIV-1 infection, DNMT2 and NSUN2 co-regulate RNA m5C modification, affecting multiple stages of viral replication (99–101). For EBV infection, NSUN2 catalyzes the m5C modification of EBER1, which impacts its metabolism and defense against the virus (102). Flaviviruses (103), hepatitis C virus (HCV) (104), Dengue Virus (DENV) (105), Zika Virus (ZIKV) (103) and others regulated by NSUN2; the methyl donor inhibitor A9145 has been found to inhibit the replication of DENV and ZIKV (105). It has been reported that the knockdown of NSUN2 does not significantly alter the m5C methylation of viral RNAs, but reduces the replication and gene expression of a variety of viruses (RSV, VSV, hMPV, SeV, and HSV) (22). In 2020, Kim et al. have selected SARS-CoV-2-infected Vero cells for their study; they uncovered the transcriptome and epitranscriptome profiles of SARS-CoV-2 for the first time using nanopore direct RNA sequencing and nanosphere DNA sequencing. However, further experiments are necessary to ascertain whether 6 (located on C atoms) of the 41 modification sites present on SARS-CoV-2 RNA include m5C modification (106). Anming Huang et al. have recently re–examined the m5C modification sites reported in HIV and MLV and analyzed the m5C methylome of SARS–CoV–2. With a rigorous bisulfite sequencing protocol and accurate data analytics though, no evidence of m5C has been found in these viruses (107). These results suggest that m5C methylation may indirectly affect SARS-CoV-2 infection.

In a recent study, Wang et al. have reported that SARS-CoV-2 infection significantly lowered NSUN2 mRNA levels in Caco-2 cells. Through transcriptome sequencing of RNA isolated from bronchoalveolar lavage fluid (BALF) of two SARS-CoV-2 patients, NSUN2 mRNA level is found decreased in SARS-CoV-2 patients compared with that in healthy individuals. In comparison to the uninfected group, the infected mice with the SARS-CoV-2 WT strain or the K18-hACE2 KI mouse model with BA.1 omicron variant-activated innate immune response show significantly lower endogenous NSUN2 mRNA levels (103). These results could be also observed in the SARS-CoV-2-infected Caco-2 cell model and SARS-CoV-2 patients, suggesting that NSUN2 plays an important regulatory role in SARS-CoV-2 infection. These findings not only offer important clues to the molecular mechanisms of m5C methylation in SARS-CoV-2 infection, but also highlight the ability of the virus to regulate gene expression in host cells. Future studies are expected to disclose the effects of these modifications on viral replication and host immune responses, opening up new avenues for the development of antiviral therapeutic strategies.

### 5.2 The role of m5C methylation in innate immunity

RNA m5C modification is crucial in the antiviral innate immune response of host cells (103) (**Figure 3**). Kariko et al. have reported that m5C-modified RNA would eliminate the activity of signaling activated by TLR3, TLR7, and TLR8. Compared with unmodified RNA, m5C-modified RNA can inhibit the expression of cytokines and DC activation markers. This indicates that m5C modification may inhibit the potential of RNA in DC activation, and lead to a weakened immune response (108). Moreover, a previous study has shown that NSUN5-mediated m5C modification of GPX4 can activate the cGAS-STING signaling (109). NSUN2, as one of the most reported “writers” of m5C modifications, is crucial in maintaining tRNA structure and regulating oxidative stress. Notably, this modification decreases the m5C level of tRNAs and the production of short and noncoding RNAs (tRF-Gln-CTG-026), which can affect the cellular translational process (110). VtRNAs formed by m5C modification can regulate gene expression, which can also be utilized by viruses to suppress the antiviral immune response of host cells (12, 14). For instance, the influenza A virus can inhibit the activation of the antiviral protein PKR through high expression of VtRNA, thereby promoting viral replication. NSUN2 may also affect the viral genome and host immune response through the regulation of RNA metabolic processing (111, 112).

NSUN2 negatively regulates IFN-I responses during multiple viral infections, including SARS-CoV-2 infection (103). During infection by RNA viruses (RSV, VSV, hMPV, and SeV) and DNA viruses (HSV), NSUN2 alters m5C levels and either directly or indirectly regulates the RIG-I signaling pathway-mediated IFN-I response through downregulating levels of specific ncRNAs (particularly RPPH1 and 7SL RNAs), thereby enhancing the antiviral response (22). In addition, NSUN2 specifically mediates m5C methylation of IRF3 mRNA and speeds up its degradation, resulting in lower levels of IRF3 and downstream IFN-β (103). NSUN2 knockout or knockdown can enhance the expression of IFN-I and downstream ISGs during various viral infections *in vitro* (103). *In vivo*, the NSUN2+/- mice show more significantly enhanced antiviral innate response than NSUN2+/+ mice. The identified highly m5C-methylated cytosines exhibit higher IRF3 mRNA levels in cells. However, SeV, VSV, HSV-1, or ZIKV infections result in reduced endogenous NSUN2 levels. In particular, SARS-CoV-2 infection (including the WT strain and the BA.1 omicron variant) also reduces the endogenous levels of NSUN2 in SARS-CoV-2 patients and K18-hACE2 KI mice, which further upregulates the levels of IFN-1 and downstream ISGs (103). These findings emphasize the significance of NSUN2 in the regulation of the host antiviral immune response. Nevertheless, there have been no published investigations on m5C modifications and IFN upstream signaling pathways. Hence, future research is anticipated to provide insights into the specific effects of these m5C modifications on viral replication and host immune responses, thus laying a solid foundation for antiviral therapeutic strategies.

## 6 Challenges and prospects

It has been recently shown that RNA m5C methylation is essential in various viral infections, including SARS-CoV-2 infection. It has been shown that 41 modified sites have been identified in SARS-CoV-2 infection, in which 6 m5C modified sites may be included. However, there has been no evidence of m5C modification in these viruses (107). This suggests that even with high-throughput sequencing technology that can offer fresh approaches, in-depth experiments are still needed to confirm the modified sites on SARS-CoV-2 RNA. Moreover, given the continuous emergence of viral variants, it is an important challenge to investigate differential m5C methylation in different SARS-CoV-2 strains, which requires global collaboration for systematic comparative studies.

m5C methylation has been recently found tightly implicated in innate immunity, which can affect RNA stabilization, nuclear export, and translation functions. For example, NSUN2 is decreased in SARS-CoV-2 infection, and high expression of NSUN2 can specifically mediate the m5C methylation of IRF3 mRNA and accelerate its degradation, leading to a low level of IRF3 production, which negatively regulates IFN-I response and affects the antiviral immune response. Additionally, m5C-modified RNA may also inhibit TLR3, TLR7, and TLR8-mediated signaling and weaken the immune response. These findings may provide clues to further explore the role of m5C methylation in SARS-CoV-2 infection and reveal new therapeutic targets for anti-SARS-CoV-2 myocarditis treatment strategies.

Although some clues about the potential role of m5C methylation in SARS-CoV-2 infection have been revealed, the specific molecular regulatory mechanism of m5C methylase, demethylase, and recognition proteins still needs further clarification, especially in the context of SARS-CoV-2 myocarditis. In the future, in-depth research is needed to explore the specific effects of m5C methylase, demethylase, and recognition proteins on viral replication and host immune response, aiming to provide novel insights into developing therapeutic strategies against SARS-CoV-2 myocarditis. Taken together, NSUN2 and m5C methylation may be promising targets for studying and treating SARS-CoV-2 and its related complications in the future.

## Author contributions

YX: Conceptualization, Data curation, Formal analysis, Funding acquisition, Writing – original draft. YL: Conceptualization, Formal analysis, Methodology, Project administration, Visualization, Writing – original draft. WQ: Data curation, Formal analysis, Writing – original draft. QZ: Conceptualization, Methodology, Project administration, Supervision, Visualization, Writing – review & editing.
